# Innovative Solutions for Patients Who Undergo Craniectomy: Protocol for a Scoping Review

**DOI:** 10.2196/50647

**Published:** 2024-03-07

**Authors:** Laura L Fernandez, Dylan Griswold, Isla Khun, Diana Victoria Rodriguez De Francisco

**Affiliations:** 1 Clinical & Translational Science Institute and Center for Global Surgery University of Utah Salt Lake City, UT United States; 2 NIHR Global Health Research Group on Neurotrauma University of Cambridge Cambridge United Kingdom; 3 Division of Neurosurgery Department of Clinical Neurosciences University of Cambridge Cambridge United Kingdom; 4 University of Cambridge Cambridge United Kingdom; 5 University of Central Florida Orlando, FL United States; 6 Universidad Metropolitana Caracas Venezuela

**Keywords:** cranioplasty, decompressive craniectomy, global neurosurgery, intellectual property, stroke, traumatic brain injury, innovative, innovative solutions, craniectomy, increased intracranial pressure, intracranial pressure, prototypes, medical devices, middle-income countries, low-income countries, noninvasive

## Abstract

**Background:**

Decompressive craniectomy (DC) is a widely used procedure to alleviate high intracranial pressure. Multidisciplinary teams have designed and implemented external medical prototypes to improve patient life quality and avoid complications following DC in patients awaiting cranioplasty (CP), including 3D printing and plaster prototypes when available.

**Objective:**

This scoping review aims to understand the extent and type of evidence about innovative external prototypes for patients who undergo DC while awaiting CP.

**Methods:**

This scoping review will use the Joanna Briggs Institute methodology for scoping reviews. This scoping review will include noninvasive medical devices for adult patients who undergo DC while waiting for CP. The search strategy will be implemented in MEDLINE, Embase, Web of Science, Scielo, Scopus, and the World Health Organization (WHO) Global Health Index Medicus. Patent documents were also allocated in Espacenet, Google Patents, and the World Intellectual Property Organization (WIPO) database.

**Results:**

This scoping review is not subject to ethical approval as there will be no involvement of patients. The dissemination plan includes publishing the review findings in a peer-reviewed journal and presenting results at conferences that engage the most pertinent stakeholders in innovation and neurosurgery.

**Conclusions:**

This scoping review will serve as a baseline to provide evidence for multidisciplinary teams currently designing these noninvasive innovations to reduce the risk of associated complications after DC, hoping that more cost-effective models can be implemented, especially in low- and middle-income countries.

**International Registered Report Identifier (IRRID):**

DERR1-10.2196/50647

## Introduction

Traumatic brain injuries (TBIs) remain a significant global health challenge affecting more than 69 million people annually [1]. The incidence of TBIs in low- and middle-income countries is disproportionate, 3 times greater than in high-income countries [1]. Severe forms of TBI (5.58 million people each year or 73 cases per 100,000 people) and other critical pathologies such as strokes and brain tumors often require a life-saving neurosurgical procedure called decompressive craniectomy (DC) [1]. DC is an essential surgical procedure that alleviates intracranial pressure, which consists of removing a section of the skull to avoid inward brain constriction due to brain parenchyma and cranial rigidity [2]. However, this intervention leaves patients vulnerable and at risk of neurological impairment, further traumas, and self-esteem difficulties while waiting for cranioplasty (CP) [3].

The overall complication rate after DC has been reported to be as high as 50% [3]. These complications include (1) the syndrome of the trephined, in which the atmospheric pressure compresses the brain parenchyma and the neurological status of the patient deteriorates after the removal of a skull bone flap, in 13% of patients who undergo DC; (2) hemorrhage, in 58%; (3) external herniation, in 25%; (4) wound complications (such as ulceration or necrosis) or surgical site infection, in up to 9%; (5) cerebral spinal fluid leakage, in 6.3%; and (6) increased risk of severe injuries from falling, among others [4-6].

A critical challenge post-DC patients face is the need for protective measures for the craniectomy site. A study described that at least 88.9% of patients have reported needing a device to prevent contact with the craniectomy site [7]. In response to this challenge, external protective devices have emerged as a potential solution to replicate the protective effects of CP and minimize postoperative complications.

Nowadays, technological innovation in medicine drives care improvements, surgical techniques, and preventative medicine. This is especially true in neurosurgery, as shown by neurostimulation in functional neurosurgery, microneurosurgery, and the merging of computed tomography and magnetic resonance imaging intraoperatively, always possible because of the multidisciplinary teams [7,8]. The global medical device industry is highly competitive, with many countries contributing to its development and growth worldwide. Interdisciplinary teams have designed and implemented external medical prototypes in response to the need to improve patient’s quality of life and avoid complications following DC in patients awaiting CP, including 3D printing and plaster prototypes, when available. Therefore, the objective of this scoping review is to develop a better understanding of available innovations in technology for patients with DC who are awaiting CP worldwide.

## Methods

### Overview

**Figure 1:**
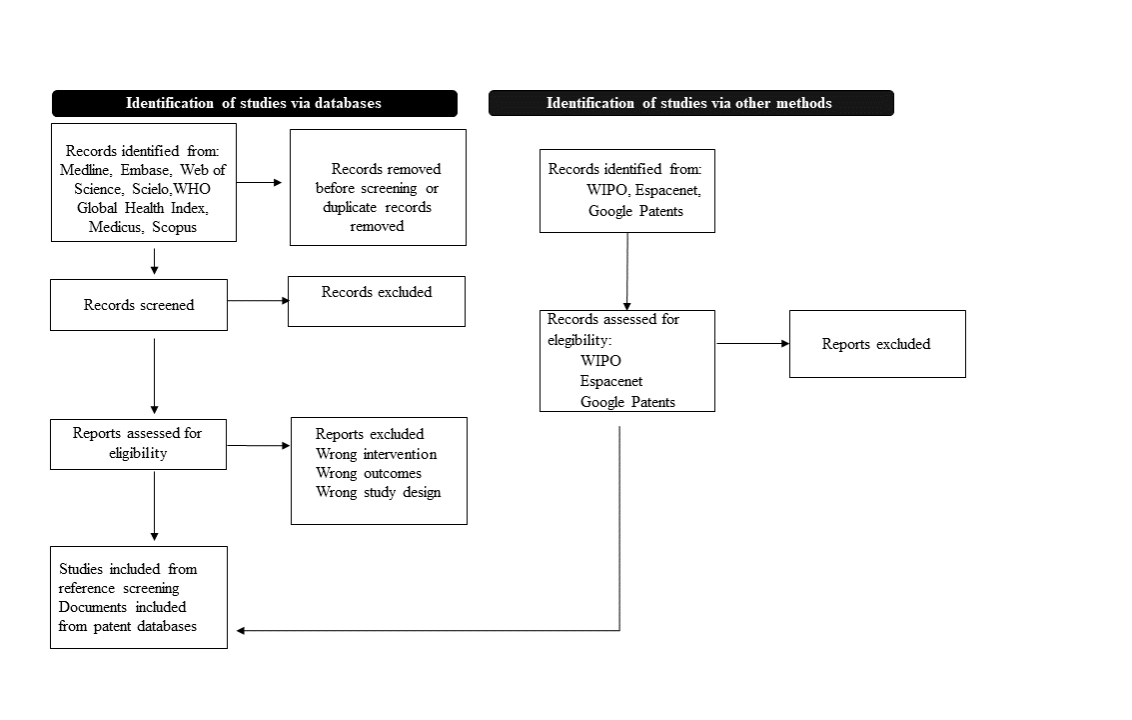
Summary of the search strategy process. WHO: World Health Organization; WIPO: World Intellectual Property Organization.

The proposed scoping review will use and follow the Joanna Briggs Institute methodology for scoping reviews [9]. The proposed methodology is presented in Figure 1.

### Review Question

What is the current landscape of external medical devices replicating the effects of CP worldwide?

### Eligibility Criteria

#### Participants

This scoping review will include prototypes for adult patients who undergo DC while waiting for CP.

#### Concept

External medical devices that replicate the effects of CP.

#### Context

This scoping review will not exclude documents based on geographic areas as it is intended to identify and locate where they have been designed and implemented.

### Types of Sources

This scoping review will consider experimental and quasi-experimental study designs, including randomized controlled trials, non-randomized controlled trials, before and after studies, and interrupted time-series studies. Consideration will be given to the inclusion of analytical observational studies, which may include prospective and retrospective cohort studies, case-control, or analytical cross-sectional studies. This review will consider descriptive observational study designs, including case series, individual case reports, and descriptive cross-sectional studies for inclusion. Qualitative studies that focus on qualitative data will also be included, but are not limited to, designs such as phenomenology, grounded theory, ethnography, qualitative description, and action research.

In addition, systematic reviews that meet the inclusion criteria will also be considered, depending on the research question. Text and opinion papers will also be considered for inclusion in this scoping review. Worldwide patents will be allocated in the central databases Espacenet, Google Patents, and World Intellectual Property Organization (WIPO).

### Search Strategy

The search strategy will be carried out by an experienced librarian (IK) to locate both published and unpublished documents. An initial limited search of MEDLINE was undertaken to identify studies on the topic. The text words in the titles and abstracts of relevant studies and the index terms used to describe the studies were used to develop a complete search strategy for PubMed, Scopus, Web of Science, Scielo (see Multimedia Appendix 1), and the World Health Organization (WHO) international database. Patents will also be allocated in Espacenet, Google Patents, and WIPO. The search strategy, including all identified keywords and index terms, will be adapted for each included database and information source. The reference list of all included sources of evidence will be screened for additional studies. Studies published in any language will be included, and translation services will be used if necessary. No time limit has been established.

### Study or Source of Evidence Selection

All identified citations will be collated and uploaded to EndNoteX9 (Clarivate Analytics) after the search. The citations will then be imported into Covidence software (Veritas Health Innovation) for screening. A total of 2 independent researchers will examine titles and abstracts for inclusion. The full text of selected studies will be retrieved and assessed. Full-text studies that do not meet the inclusion criteria will be excluded, and the reasons for exclusion will be provided in the final scoping review. Any disagreements between the researchers during either title and abstract screening or full-text screening will be resolved through discussion or with a third reviewer. All included studies will undergo a process of data extraction using a standardized data extraction tool. The final study will report the search results in complete and present using the Preferred Reporting Items for Systematic Reviews and Meta-Analyses extension for Scoping Reviews (PRISMA-ScR) checklist.

### Data Extraction

Data will be extracted from papers and patent documents included in the scoping review by 2 or more independent reviewers using a data extraction tool developed by the reviewers. The data extracted will include specific details about the participants, concept, context, study methods, and critical findings relevant to the review questions.

A draft extraction is provided in Multimedia Appendix 2. It will be modified as necessary while extracting data from each included evidence source. Modifications will be detailed in the scoping review. Any reviewer disagreements will be resolved through discussion or with additional reviewers. If appropriate, authors of studies could be contacted to request missing or other data, where required.

### Data Analysis and Presentation

The extracted data will be presented in tabular form and as a narrative summary that aligns with the aim of this scoping review. The table will report (1) the distribution of medical devices by countries of origin or study design, (2) functional claims or features, (3) implementation strategies, (4) patient outcomes, (5) costs, and (6) strengths and weaknesses. This table may be further refined at the review stage. Graphical representations may be used, including bar charts, line charts, pie charts, and diagrams. A narrative summary will accompany the tabulated or charted results and describe how the results relate to the review’s objectives.

### Ethical Considerations

No ethics approval will be required, as this review is based on already published data and does not involve interaction with human participants.

## Results

The research for this systematic review commenced in February 2023, and we expect to publish the findings in 2024. The plan for dissemination, however, is to publish the review results in a peer-reviewed journal and present findings at high-level conferences that engage the most pertinent stakeholders involved in innovation and neurosurgery.

## Discussion

Cumulatively, 6838 studies were identified from the database searches and 1652 from patent searches, after which 64 scientific papers and 4 patent documents were left for full-text review. Of these, 9 documents met the inclusion criteria and will be used in the final synthesis. Three categories of study design were identified: case reports (n=6), cohort studies (n=1), and exploratory clinical trials (n=1). One of the documents was not a scientific report and was categorized as a patent document. The complete data analysis and discussion will be published in an indexed journal once it is finished in an indexed journal upon completion.

This protocol has been rigorously developed and explicitly designed to illustrate and summarize the evidence regarding innovative external devices for patients with DC awaiting CP worldwide. This scoping review will serve as a baseline to provide evidence for multidisciplinary teams currently designing these noninvasive innovations to reduce the risk of associated complications after DC, hoping that more cost-effective models can be implemented, especially in middle-income and low-income countries. The principal limitation of this and any scoping review is the quality of the included studies leaving the included literature with a higher risk of bias.

Although most of these devices were developed in middle- and high-income countries, they serve as a starting point for implementation and future development in lower-income countries where no current solutions exist.

## Appendix

Multimedia Appendix 1 Pubmed and Scopus Search strategy.

Multimedia Appendix 2 Data extraction instrument.

## Abbreviations

CP: cranioplasty
DC: decompressive craniectomy
PRISMA-ScR: Preferred Reporting Items for Systematic Reviews and Meta-Analyses extension for Scoping Reviews
TBI: traumatic brain injury
WHO: World Health Organization
WIPO: World Intellectual Property Organization

## Data Availability

The data sets generated and analyzed during this study are available from the corresponding author on reasonable request.
