# Supplementary material for: Innovative Solutions for Patients Who Undergo Craniectomy: Protocol for a Scoping Review
Source: JMIR Res Protoc. 2024 Mar 7;13:e50647. doi: 10.2196/50647 (PMC10958337; doi:10.2196/50647)
Supplement: Multimedia Appendix 1 [file resprot_v13i1e50647_app1.docx]

| **Electronic Database** | **Query** | **Records retrieved** |
| --- | --- | --- |
| Medline | 1 (external cranial plate* or ((3d print* or three dimensional print*) adj2 (helmet* or plate*)) or plaster cast* or prosthe* or external cranial rotection or head protection or (helmet adj3 protect*) or external device* or temporary treatment* or prototype* or (patient specific adj (mould* or device* or reconstruction*)) or manufactured mold* or light scanned model* or silicone mold* or (custom* adj (pmma or poly* or cranioplast*)) or ((hydro* or titanium* or ceramic* or cranial* or custom* or cranioplast* or patient specific or poly* or silicone*) adj3 implant*)).ti,ab,kw,kf. or casts, surgical/ or exp &quot;Prostheses and Implants&quot;/ 710305  2 ((plastic surgery or reconstructive surgery).ti,ab,kw,kf. or plastic surgery procedures/ or surgery, plastic/) and (material* or timing).ti,ab,kw,kf. 9165  3 (trephin* or craniectom* or craniotom* or trepan*).ti,ab,kw,kf. or exp trephining/ or exp craniotomy/ or exp Decompressive Craniectomy/ 35121  4 3 and (1 or 2) 2051 | 2051 |
| Scopus | ( TITLE-ABS-KEY ( &quot;external cranial plate*&quot;  OR  ( ( &quot;3d print*&quot;  OR  &quot;three dimensional rint*&quot; )  W/2  ( helmet*  OR  plate* ) )  OR  &quot;plaster cast*&quot;  OR  prosthe*  OR  &quot;external cranial protection&quot;  OR  &quot;head protection&quot;  OR  ( helmet  W/3  protect* )  OR  &quot;external device*&quot;  OR  &quot;temporary treatment*&quot;  OR  prototype*  OR  ( &quot;patient pecific&quot;  W/1  ( mould*  OR  device*  OR  reconstruction* ) )  OR  &quot;manufactured mold*&quot;  OR  &quot;light scanned model*&quot;  OR  &quot;silicone mold*&quot;  OR  ( custom*  W/1  ( pmma  OR  poly*  OR  cranioplast* ) )  OR  ( ( hydro*  OR  titanium*  OR  ceramic*  OR  cranial*  OR  custom*  OR  cranioplast*  OR  &quot;patient specific&quot;  OR  poly*  OR  silicone* )  W/3  implant* ) )  OR  TITLE-ABS-KEY ( ( &quot;plastic surgery&quot;  OR  &quot;reconstructive surgery&quot;  OR  cranioplast* )  AND  ( material*  OR  timing ) ) )  AND  ( TITLE-ABS- KEY ( trephin*  OR  craniectom*  OR  craniotom*  OR  trepan* ) ) | 2157 |
