# Supplementary material for: Innovative Solutions for Patients Who Undergo Craniectomy: Protocol for a Scoping Review
Source: JMIR Res Protoc. 2024 Mar 7;13:e50647. doi: 10.2196/50647 (PMC10958337; doi:10.2196/50647)
Supplement: Multimedia Appendix 2 [file resprot_v13i1e50647_app2.docx]

| Title | Title of the manuscript or medical device |
| --- | --- |
| Author/ Inventor(s) |  |
| Publication Type / study design | Scientific paper, utility patent, etc. |
| Product | Type of medical device (helmet, plate, etc) |
| Date of publication and lead institution country | - |
| Field(s) of expertise | Neurosurgery, plastic surgery, engineering, etc. |
| The intended country for implementation | Different from the country of publication |
| Materials or Design claims/ features | The matter from which a thing is or can be made |
| Patient-specific (Y/N) | Design for each patient’s condition |
| Device classification (FDA, CE, etc) | - |
| Functional claims/features | - |
| Cost (USD) | Production costs, etc. |
| Tested in patients (Y/N/Unknown) | - |
| Patient characteristics | - |
| Patient outcomes | - |
| Funding sources | - |
